# Metapopulation Structure of Two Species of Pikeworm (*Triaenophorus*, Cestoda) Parasitizing the Postglacial Fish Community in an Oligotrophic Lake

**DOI:** 10.3390/ani13193122

**Published:** 2023-10-06

**Authors:** Elena N. Kashinskaya, Pavel G. Vlasenko, Tatyana V. Kolmogorova, Gelena V. Izotova, Anastasiya V. Shokurova, Georgy A. Romanenko, Grigorii N. Markevich, Karl B. Andree, Mikhail M. Solovyev

**Affiliations:** 1 Papanin Institute for Biology of Inland Waters, Russian Academy of Sciences, Borok 152742, Yaroslavl Region, Russia; 2 Institute of Systematics and Ecology of Animals of Siberian Branch, Russian Academy of Sciences, Novosibirsk 630091, Russiayarmak85@mail.ru (M.M.S.); 3 A.N. Severtsov Institute of Ecology and Evolution, Russian Academy of Sciences, Moscow 119071, Russia; 4 Altai Branch of «VNIRO» («AltaiNIRO»), Barnaul 656043, Russia; 5 FBUZ «Center of Hygiene and Epidemiology in the Altai Territory», Barnaul 656049, Russia; 6 Kronotsky Nature Reserve, Yelizovo 684000, Kamchatka Region, Russia; 7 Institut de Recerca i Tecnologìa Agroalimentaries (IRTA), 43540 Sant Carles de la Ràpita, Spain; 8 Biological Institute, Tomsk State University, Tomsk 634050, Russia

**Keywords:** tapeworm, cestodes, *Triaenophorus crassus*, *Triaenophorus nodulosus*, food web, trophic interactions, intermediate host, definitive host, Siberia, postglacial fish

## Abstract

**Simple Summary:**

In this study, the levels of infestation of two different species of pikeworm, *Triaenophorus crassus* and *T. nodulosus*, inhabiting a deep, oligotrophic mountain lake in Siberia were revealed. Based on results of five years of studies, a significant interannual variation in *T. crassus* infestation level was found, whereas the stability of *T. nodulosus* infestation in their host fishes was seen. Differences in feeding habits and physiology of fishesprobably had an effect on the mass of parasite abundance. Moreover, an asymmetry in parasite infestations between the number of *T. crassus* cysts in the left and right body surfaces of the ‘‘benthivorous” *Coregonus lavaretus pidschian* was also observed.

**Abstract:**

In the present study, we estimated the levels of infestation of the main fish species that are hosts for two *Triaenophorus* species: *T. crassus* and *T. nodulosus*. The prevalence of *T. crassus* and *T. nodulosus* infestations in the intestine of their definitive host–pike *Esox lucius* was similar (71.0% and 77.4%, respectively). At the same time, the prevalence of *T. crassus* infestation in muscle tissue was significantly different between the second intermediate hosts, *Coregonus lavaretus pidschian* (31.4%) and *Cor. l. pravdinellus* (91.2%), due to considerable differences in their diets. For *T. nodulosus*, we found significant variations in the levels of prevalence among the second intermediate hosts—100% for *Lota lota*, 81.8% for *Cottus sibiricus* 31.9% for *Thymallus arcticus*, and 24.5% for *Perca fluviatilis*—that we also explained using different diets. Moreover, analysis of the symmetry of parasite infestations did not reveal any asymmetry between the number of cysts in the left and right body surfaces of the “planktivorous” form/species of whitefish, whereas in the ‘‘benthivorous”, an asymmetry of parasite infestations was found.

## 1. Introduction

The majority of helminths have complex lifecycles that can include one or several intermediate hosts and are transmitted through food webs [1]. Different fish species are characterized by very diverse feeding habits and strategies while occupying different positions in aquatic food webs. At the same time, fishes may consume a plethora of different species of invertebrates that are common first intermediate hosts for helminths [1]. Due to the species-specific ranges of hosts for various helminths and different feeding habits among fish species, the parasites found in or on fish are used as one of the trophic indicators that may clarify the trophic relationships among species in aquatic food webs. The role of parasites as indicators of trophic diversification of fish has been discussed in recent studies [1,2,3,4]. In general, a high level of fish infestation positively correlates with high consumption of infected food items that are the first intermediate host for transmitted helminths. Moreover, the specific parasite found in a fish host may indicate that this fish consumes specific food items, even if this food item was never registered by other approaches like direct observation of gut content [4].

Tapeworms of the genus *Triaenophorus* (Cestoda, Bothriocephalidea: Triaenophoridae) are widespread and highly specialized parasites of Holarctic fish. According to a recent study based on sequencing of partial cox1 mtDNA and nuclear 28S rRNA genes, the Eurasian pikeworm *Triaenophorus* spp. consists of five valid species (*T. amurensis* Kuperman, 1968; *T. crassus* Forel, 1868; *T. meridionalis* Kuperman, 1968; *T. nodulosus* Pallas, 1781, Rudolphi, 1793; and *T. orientalis* Kuperman, 1968) [5]. The life cycle of these cestodes is complex and proceeds with the passage through two intermediate hosts and one definitive host. Cestodes become sexually mature only in the intestines of pike *Esox lucius* Linnaeus, 1758 after feeding on fishes infected with these cestodes [6].

Infestations with plerocercoids and adults of different *Triaenophorus* species are particularly common in freshwater fishes. The geographic distribution of the *Triaenophorus* species are circumpolar and includes more than 70 fish species that have been described as possible second intermediate and definitive hosts [5,6,7]. For freshwater bodies of West Siberia (Russia), two species, *T. crassus* and *T. nodulosus*, are of epizootic importance. *Triaenophorus* cestodes do not present a health hazard to humans, but these parasites can cause financial damage to fisheries due to the unappetizing appearance of infested fish. *T. crassus* and *T. nodulosus* differ in morphological features and localization in the second intermediate hosts. The main morphological differences of *T. crassus* from *T. nodulosus* are the shape of the scolex hooks, which have a massive basal part and small straight prongs [5,6]. The number of second intermediate hosts for *T. crassus* is much lower than for *T. nodulosus*; some hosts are common to both species. The plerocercoid of *T. crassus* is localized in the musculature of fish from the subfamilies Salmoninae and Coregoninae, while *T. nodulosus* is localized in the liver of Percidae, Thimallinae, Cobitidae, Osmeridae, Cyprinidae, Cottiidae, Lotidae, and several other families and subfamilies of fishes [6]. 

Several fish species inhabiting Teletskoye Lake (Altai Republic, Western Siberia, Russia) are known as common hosts for these two *Triaenophorus* species. Thus, *T. nodulosus* is commonly found in the liver of Siberian sculpin *Cottus sibiricus* Kessler, 1889; perch *Perca fluviatilis* Linnaeus, 1758; arctic grayling *Thymallus arcticus* Pallas, 1776; and burbot *Lota lota* Linnaeus, 1758 [5,8]. While *T. crassus* typically parasitizes muscle tissue of a sympatric pair of coregonid whitefishes—the planktivorous “dwarf” form/species named *Coregonus lavaretus pravdinellus* Dulkeit, 1949 and the benthivorous “normal” form/species named *Cor. l. pidschian* Gmelin, 1789 [5,9]. The definitive host for both *Triaenophorus* species in the lake is pike, *Esox lucius* Linnaeus, 1758 where these helminths are found in the intestine. 

Teletskoye Lake is one of the biggest montane, oligotrophic, deep-water bodies located in the south of Siberia that is a refugium since the last glaciations for several fish species including endemic whitefish—*Cor. l. pravdinellus*. Although the main host ranges are known for both *Triaenophorus* species in this lake, previous studies have provided very limited information regarding the ecological features of these parasites such as transmissions in the food web of the lake, distribution of parasites in different parts of the fish body, interannual variability of infestation levels, etc.

Thus, the main aim of the present study was to estimate the infestation levels of both *Triaenophorus* species in the second intermediate and the definitive hosts from Teletskoye Lake. We tested the following three hypotheses: (1) the main driver that determines the levels of infestation in second and definitive hosts is feeding habits; this factor does not directly reflect the expected route of parasite transmission, e.g., planktonic crustaceans (first intermediate host), planktivorous fish (second intermediate host), pike (definitive host); (2) the infestation levels of pike, as the definitive host, will be different for each *Triaenophorus* species due to the studied *Triaenophorus* species having different life strategies: *T. crassus* infects only two secondary host fish species, whereas *T. nodulosus* is able to infect at least six fish species as the second intermediate host; and (3) the body mass of the studied *Triaenophorus* plerocercoids will be different in various second intermediate hosts.

## 2. Materials and Methods

### 2.1. Sample Collection 

**Figure 1:**
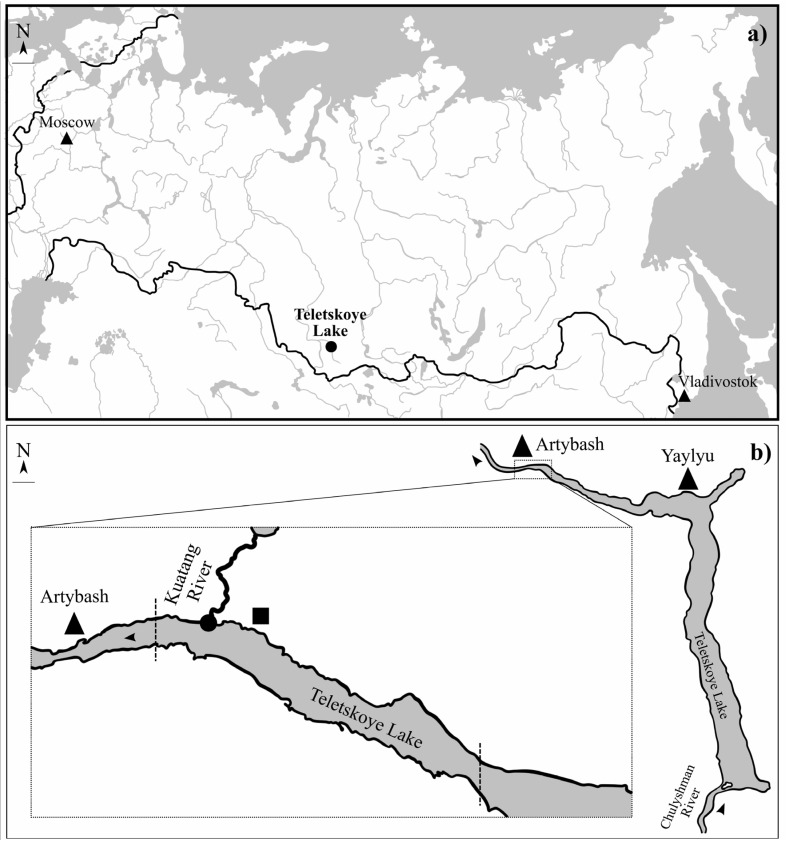
General map of Russian Federation (**a**) and map of the Teletskoye Lake with sampling point and area (**b**): dot—sampling point of *Phoxinus phoxinus*; dotted lines—sampling area of other fishes; triangle—village; rectangle—field station; arrow—water flow direction.

A total of 507 fishes, belonging to (Coregoninae, Cottidae, Percidae, Gadidae, Cyprinidae were investigated (Table 1). The fishes (with exception common minnow *Phoxinus phoxinus* Linnaeus, 1758) were collected at the end of August and the end of October between 2019 and 2022 in the north part of Teletskoye Lake (51°79′ N; 87°30′ E). Teletskoye Lake is a large (223 km^2^) and deep (maximum 325 m) oligotrophic lake (basin of Ob River) in the Altai Mountains (Altai Republic, Russia). *Ph. phoxinus* was collected from a small river, Kuatang, that flows into Teletskoye Lake. Fish from the lake were captured using gill nets (mesh sizes 18–60 mm) at depths from 2 to 40 m. *Ph. Phoxinus* was collected using a fish net at a depth 0.5 m (Figure 1). After capture, all fish were transported to the laboratory in the Teletskoye Lake field station of the Institute of Systematic and Ecology of Animals SB RAS. 

The fishes were humanely sacrificed by a blow to the head before sampling. Afterwards, the fishes were identified, measured (total length, TL), and weighed (total body weight, or “BW”, and eviscerated body weight, or “EBW”). Male and female fishes were identified according to gonadal development. The total number of fishes collected for study included: 140 “normal” whitefish *Cor. l. pidschian* and 89 “dwarf” whitefish *Cor. l. pravdinellus*, 55 Siberian sculpin *Cot. sibiricus*, 102 perch *Pe. fluviatilis*, 47 arctic grayling *Th. arcticus*, 35 burbot *L. lota*, 8 common minnow *Ph. phoxinus*, and 31 pike *E. lucius*. 

### 2.2. Parasitological Analysis

Plerocercoids were removed from cysts and stored in 70% ethanol. The scolex hooks were measured on the squashed scolices mounted in Berlese’s medium. The species identification of the cestodes was performed in accordance with identification keys using characters described earlier [6,10]. The prevalence and mean intensity of parasite infestation were calculated according to the standard definitions described by Bush et al. [11]. In addition, the wet weight of plerocercoids/adult worms and wet weight of fish livers and intestines (*E. lucius*) were measured using an electronic scale SCOUT™ STX (Göteborg, Sweden). The ratio of the wet weight of plerocercoids/adult worm from wet weight of infected organ (liver or intestine), total body weight of fish and eviscerated body weight of fish were calculated. 

### 2.3. Statistical Analysis 

All data are presented as a mean ± standard error (SE). To estimate the differences between parasite intensity and abundance across different sampling years (2019–2022), as well as in terms of fish total length and body weight among different sampling years, the Tukey HSD post-hoc test was applied using PAST v. 3.16 [12]. In the same software, to explore the effect of gender (sex) as a factor on the parasite abundance and intensity, this factor was tested using one-way ANOVA with statistical significance established at *p* < 0.05. To estimate the level of correlations between parasite intensity, abundance and fish total length, total body weight, and gender (sex), a Spearman rank correlation test (ρ) was used. The relationship between the number of *T. crassus* and *T. nodulosus* individuals and the total length or total body weight of definitive host—*E. lucius*—were estimated using the linear regression realized in Statistica 8.0. In the same software, the relationship between the weight of *T. crassus* and *T. nodulosus* and number of cysts in different intermediate hosts were estimated. Differences of slopes of two regression lines in terms of total length and body weight of *E. lucius* were calculated using the Student’s *t*-test implemented in Excel MS 2016. Density plots were created using ggplot2 R package [13]. To characterize the distribution, the aggregation indices were calculated using QPweb Version 1.0.15 [14]. To analyze the symmetry of *T. crassus* infestations in different forms/species of whitefishes *Coregonus lavaretus* a binomial exact test was performed using aspi R package in R software version 4.1.2 (R Core Team, 2021) [15].

## 3. Results

### 3.1. Prevalence and Intensity of Fish Infestation with Triaenophorus spp.

#### 3.1.1. Intermediate Hosts Infected by *T. crassus*

*T. crassus* cysts was found in muscle of both forms/species of whitefishes *Coregonus lavaretus*. A single cyst from muscle contained one plerocercoid. The prevalence of *T. crassus* infestation in the muscles of the “dwarf” whitefish *Cor. l. pravdinellus* was higher than that in the muscles of the ‘‘normal” whitefish *Cor. l. pidschian* for all studied years and ranged from 89.7 to 100% and from 27.3 to 41.5%, respectively (Table 2). 

The mean intensity and abundance of parasite infestation in the muscles of the “dwarf” whitefish *Cor. l. pravdinellus* ranged from 4.5 ± 0.5 to 5.0 ± 0.8 worms and from 4.0 ± 0.5 to 5.0 ± 0.8 worms, respectively, whereas the same indices of parasite infestation in the muscles of the “normal” whitefish *Cor. l. pidschian* ranged from 1.8 ± 0.4 to 1.9 ± 0.5 worms and from 0.5 ± 0.2 to 0.8 ± 0.2 worms, respectively (Table 2). 

**Figure 2:**
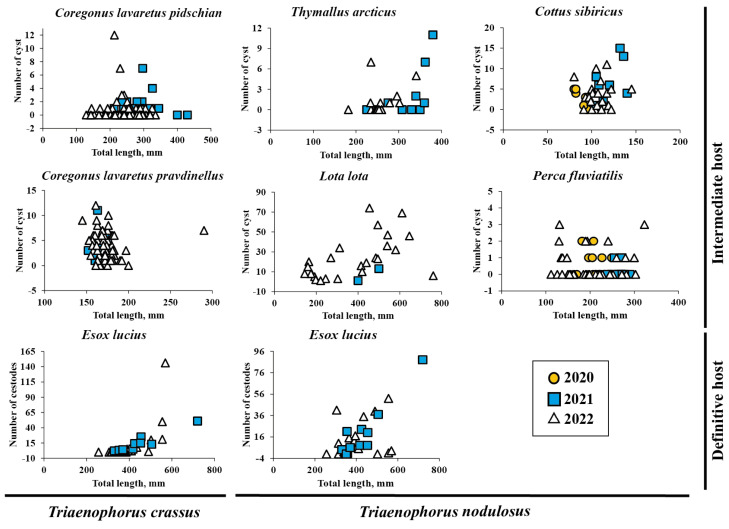
The abundance of *Triaenophorus crassus* and *T. nodulosus* contrasted with total length of different intermediate and definitive hosts from Teletskoye Lake from 2020 to 2022.

The abundance of *T. crassus* compared with the total length of studied fishes is shown in Figure 2. There was no difference in the mean intensity and abundance of *T. crassus* infecting the muscles of both forms/species of whitefishes *Coregonus lavaretus* between the studied years, 2021 and 2022 (“dwarf”: ANOVA (intensity), F = 0.28, df = 35.8, *p* = 0.60, ANOVA (abundance) F = 1.1, df = 34.6, *p* = 0.31; ‘‘normal”: ANOVA (intensity), F = 0.002, df = 41.9, *p* = 0.96, ANOVA (abundance) F = 0.96, df = 82.2, *p* = 0.33). 

A positive significant correlation was found between total body weight of the “dwarf” whitefish *Cor. l. pravdinellus* and the number of cysts in muscle tissue in 2021 (Spearman ρ = 0.55, *p* = 0.01, *n* = 20). In other cases, no statistically significant correlations were found between the number of cysts in muscle and both length and weight of “dwarf” and ‘‘normal” forms/species of whitefishes *Coregonus lavaretus*.

The factor “sex” had no effect on the number of cysts *T. crassus* in fish muscles (“dwarf”: Spearman ρ = −0.05, *p* = 0.86, *n* = 16, 2021; Spearman ρ = −0.11, *p* = 0.43, *n* = 52, 2022; ‘‘normal”: Spearman ρ = 0.07, *p* = 0.21, *n* = 30, 2021; Spearman ρ = −0.19, *p* = 0.08, *n* = 83, 2022).

#### 3.1.2. Intermediate Hosts Infected by *T. nodulosus*

*T. nodulosus* was identified in the liver of *Cot. sibiricus*, *Pe. fluviatilis*, *Th. arcticus*, and *L. lota*. A single cyst from the liver contained one plerocercoid. In only one fish species (*Ph. phoxinus*), were there no plerocercoids found in their liver. The prevalence of *T. nodulosus* infestation in the liver of *L. lota* (100% for years 2021 and 2022) was higher than that in the liver of *Cot. sibiricus*, *Th. arcticus*, *Pe. fluviatilis*, where the prevalence level ranged from 66.7 to 100%, 30.4 to 33.3%, and 20.0 to 27.3%, respectively, for those same years (Table 2). The mean intensity of parasite infestation in the liver of *L. lota*, *Cot. sibiricus*, *Th. arcticus*, and *Pe. fluviatilis* ranged from 7.4 ± 2.3 to 23.5 ± 3.7, 3.6 ± 0.8 to 8.0 ± 2.3, 2.6 ± 0.8 to 4.1 ± 1.3, and 1.0 ± 0.0 to 1.8 ± 0.2, correspondingly (Table 2). 

The mean abundance of parasite infestation in the liver of *L. lota*, *Cot. sibiricus*, *Th. arcticus*, and *Pe. fluviatilis* ranged from 7.4 ± 2.3 to 23.5 ± 3.7, 2.4 ± 0.8 to 8.0 ± 2.3, 0.8 ± 0.1 to 1.4 ± 0.6, and 0.2 ± 0.1 to 0.5 ± 0.1, respectively. The abundance of *T. nodulosus* compared with the total length of studied fishes is shown in Figure 2. The mean intensity and abundance of *T. nodulosus* infecting the liver of fishes were not significantly different between studied years (2019–2022). There was one exception for *Cot. sibiricus*, where the significant differences in mean abundance was obtained between the years 2020 (8.0 ± 2.3) and 2021 (3.3 ± 0.6) (ANOVA, Tukey HSD post-hoc test, F = 4.2, df = 3, *p* = 0.013) and between the years 2021 and 2022 (2.4 ± 0.8) (ANOVA, Tukey HSD post-hoc test, F = 4.2, df = 3, *p* = 0.015). 

A positive significant correlation was found between *L. lota* total body weight/total length and the number of cysts in its liver in 2022 (total length: Spearman ρ = 0.61, *p* = 0.0006, *n* = 30), (total weight: Spearman ρ = 0.60, *p* = 0.002, *n* = 25). A positive significant correlation was also found between total length of the *Th. arcticus* and the number of cysts in its liver in 2021 (Spearman ρ = 0.54, *p* = 0.020, *n* = 19). Throughout all of the studied years, a positive significant correlation was found between the total length/weight of the *Th. arcticus* and the number of cysts (total length: Spearman ρ = 0.37, *p* = 0.020, *n* = 38; total weight: Spearman ρ = 0.39, *p* = 0.019, *n* = 36). In other cases, there were no statistically significant correlations found between the number of cysts in liver and both length and weight of fishes. The factor “sex” had no effect on the intensity and abundance of *T. nodulosus* in fish liver. 

#### 3.1.3. Distribution of *T. crassus* and *T. nodulosus* Plerocercoids

**Figure 3:**
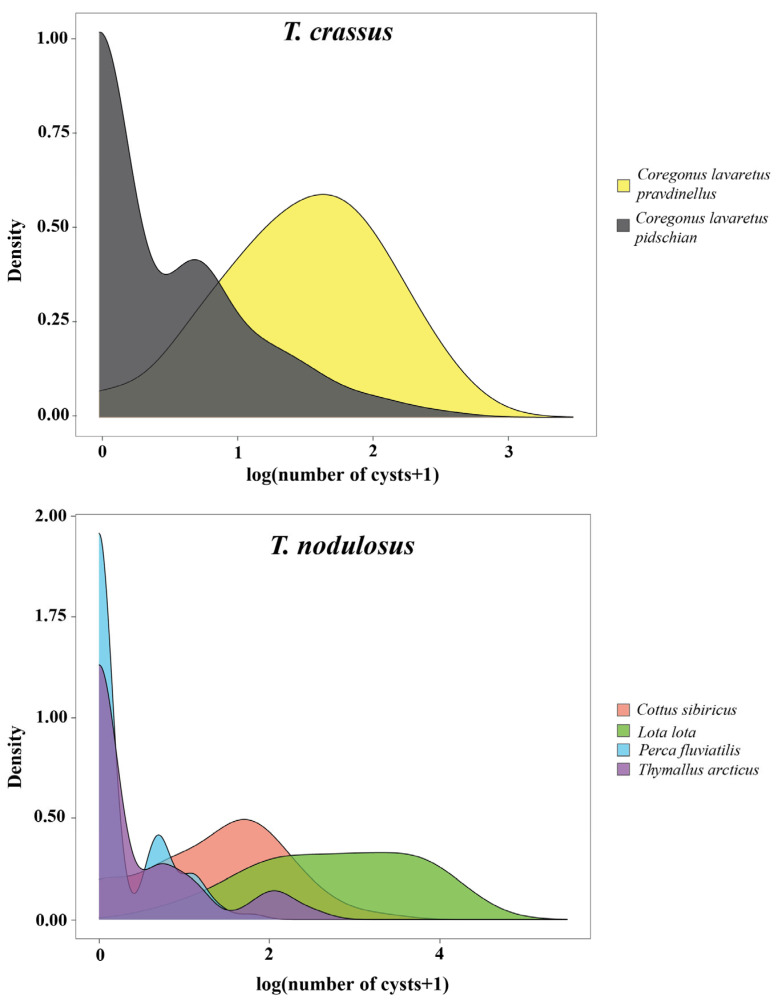
Distribution plot of the number of *T. crassus* and *T. nodulosus* plerocercoids per host species.

The distribution patterns of *T. crassus* plerocercoids in two forms/species of whitefishes *Coregonus lavaretus* were strictly different (Figure 3). Parasite aggregation was significantly higher for *Cor. l. pidschian* in comparison with *Cor. l. pravdinellus* (Poulin’s discrepancy index were amounted to 0.756 and 0.391, respectively). The variance/mean ratio differed to a lesser extent and amounted to 3.17 and 2.70, respectively. *Cor. l. pidschian* infestations were characterized by the presence of a large number of uninfected individuals and at low intensity, while plerocercoids from *Cor. l. pravdinellus* were more evenly distributed (Figure 3). 

The highest Poulin’s discrepancy index for *T. nodulosus* was shown for *Th. arcticus* (0.809), the lowest one for *L. lota* (0.454). The variance/mean ratio was minimal in *Pe. fluviatilis* and maximal in *L. lota* (1.71 and 17.36, respectively). The shape of distribution of *T. nodulosus* was similar for both *Pe. fluviatilis* and *Th. arcticus*. *Cot. sibiricus* and *L. lota* were characterized by a small number or absence of uninfected individuals and higher values of intensity. In general, the variance/mean ratio values of more than one indicated an aggregation of plerocercoids among hosts and preclude a random or uniform distribution.

#### 3.1.4. Definitive Host

The prevalence of *T. crassus* infestation in the intestine of *E. lucius* was higher in 2022 than that in 2021 and amounted to 78.6 and 64.7%, respectively, whereas the prevalence of *T. nodulosus* in the intestine of *E. lucius* did not change during the studied years (76.5 and 78.6% for 2021 and 2022 years) (Table 2). The mean intensity and abundance of *T. crassus* infestation in the intestine of *E. lucius* ranged from 12.3 ± 5.6 to 23.6 ± 6.3 worms and 9.6 ± 3.8 to 15.3 ± 8.8 worms, respectively, whereas the mean intensity and abundance of *T. nodulosus* ranged from 18.8 ± 4.8 to 19.7 ± 6.8 worms and 14.4 ± 4.1 to 15.5 ± 6.3 worms, respectively (Table 2). The mean intensity and abundance of *T. crassus* and *T. nodulosus* infecting the intestine of *E. lucius* were not different between studied years (ANOVA (intensity), F = 0.68, df = 1.0, *p* = 0.42, *T. crassus*; ANOVA (abundance), F = 0.3, df = 1.0, *p* = 0.59, *T. crassus*; ANOVA (intensity), F = 0.01, df = 1.0, *p* = 0.91, *T. nodulosus*; ANOVA (abundance), F = 0.03, df = 1.0, *p* = 0.88, *T. nodulosus*).

**Figure 4:**
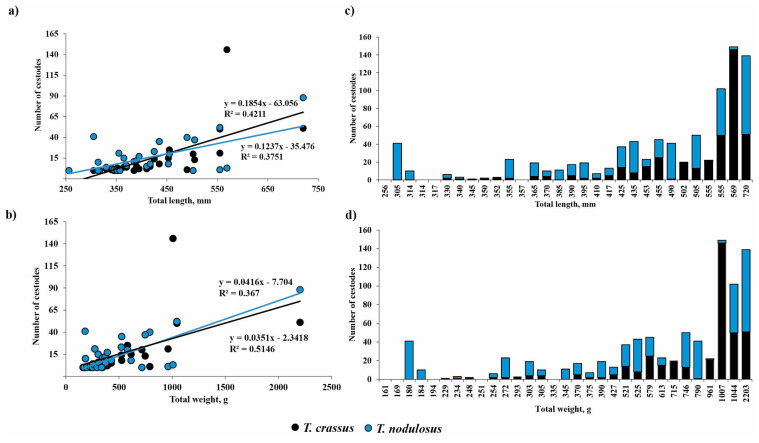
Relationship between the number of *Triaenophorus crassus* and *T. nodulosus* and total length (**a**,**c**) or total body weight (**b**,**d**) of definitive host—*Esox lucius*—from Teletskoye Lake during studied years (2021, 2022). The black and blue lines on Figure (**a**,**b**) show the linear regression with R-squared for *T. crassus* and *T. nodulosus*, correspondingly.

Mean abundance of *T. crassus* in the intestine of *E. lucius* significantly increased with increasing total length and weight of fish (Figure 4, TL: R^2^ = 0.42, F = 0.0001, *n* = 30; TW: R^2^ = 0.37, F = 0.0003, *n* = 31). There are no differences for the slopes of the two regression lines in terms of length and weight of fish. 

No sex-related differences of mean abundance (R^2^ = 0.034, F = 0.397, *n* = 23) were found for *T. crassus* in the intestine of *E. lucius*. The mean abundance of *T. nodulosus* in *E. lucius* intestine had also significantly increased with fish total length and total bodyweight (Figure 4a,b, TL: R^2^ = 0.38, F = 0.0003, *n* = 30; TW: R^2^ = 0.51, F = 5.59 × 10^−6^, *n* = 31). A significant relationship was also found between mean abundance of *T. nodulosus* and gender (sex) of *E. lucius* (R^2^ = 0.28, F = 0.009, *n* = 23). In the group of fish with total length from 250 to 555 mm and total body weight from 150 to 800 g, the number of *T. nodulosus* in the intestine of *E. lucius* was higher in comparison with number of *T. crassus*, whereas in the larger fish group (TL and TW ranged from 555 to 1000 mm and from 1000 to 2300 g, respectively), the ratio of *T. crassus* was replaced by *T. nodulosus* and reached a maximum of 146 and 88 cestodes in one pike specimen (Figure 4c,d).

### 3.2. The Relationship between Wet Weight of Cestodes and Their Hosts

#### 3.2.1. Intermediate Hosts Infected by *T. crassus*

**Figure 5:**
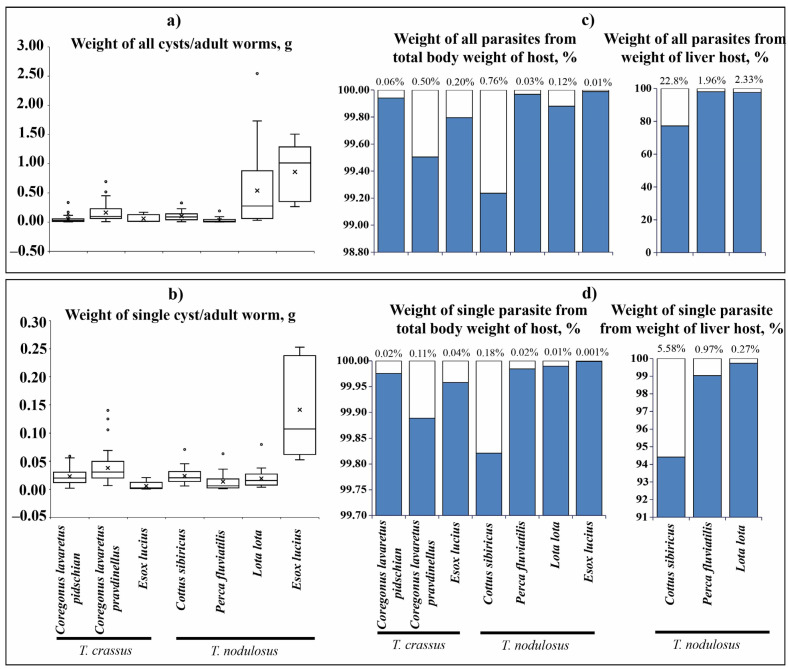
Size of cestodes (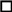) and their relationship with fish total body weight (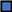). (**a**) Weight of all cysts or adult worms, g; (**b**) weight of single cyst or adult worm, g; (**c**) weight of all cysts or adult worms from total body weight, and weight of liver of fish, %; (**d**) weight of single cyst or adult worm from total body weight, and weight of liver of fish, %. Boxplots represent mean (crosses) with outliers (empty circles). The percent on the top of graph (**c**,**d**) indicates the weight of parasites.

The total wet weight of *T. crassus* plerocercoids infecting the muscles of both forms/species of whitefishes *Coregonus lavaretus* ranged from 0.002 to 0.693 g (Figure 5a). The mean wet weight of all *T. crassus* plerocercoids from muscle of the “dwarf” form/species *Cor. l. pravdinellus* (0.161 ± 0.019 g) was significantly higher if compared to the ‘‘normal” whitefish *Cor. l. pidschian* (0.054 ± 0.011 g) (ANOVA, F = 17.1, df = 1.0, *p* = 0.00008). The mean wet weight of individual specimens of *T. crassus* plerocercoids from muscles of “dwarf” form/species *Cor. l. pravdinellus* (0.04 ± 0.002 g) was also significantly higher when compared to the ‘‘normal” (0.02 ± 0.003 g) whitefish *Cor. l. pidschian* (ANOVA, F = 10.1, df = 1.0, *p* = 0.002) (Figure 5b). The wet weight of *T. crassus* plerocercoids did not exceed one percent of the host total weight and weight eviscerated (Figure 5c,d; Figure S1). A positive significant correlation was found between the number of *T. crassus* cysts and their mean wet weight of cysts. In case of the average individual wet weight of cysts and number of cysts, there were no statistically significant correlations found (Table 3).

#### 3.2.2. Intermediate Hosts Infected by *T. nodulosus*

The total wet weight of *T. nodulosus* infecting the liver of intermediate hosts (*Cot. sibiricus*, *L. lota*, and *Pe. fluviatilis*) ranged from 0.001 to 2.543 g (Figure 5a). For *T. nodulosus* from different hosts, the highest total wet weight of plerocercoids was registered from *L. lota* (0.538 ± 0.111 g) in comparison with *Cot. sibiricus* (0.100 ± 0.015 g), and *Pe. fluviatilis* (0.035 ± 0.014 g) (ANOVA, Tukey HSD post-hoc test, F = 10.7, df = 65.0, *p* < 0.05). The highest individual weight of *T. nodulosus* was registered from the liver of *Cot. sibiricus* (0.024 ± 0.003), whereas from *L. lota* (0.019 ± 0.002 g) and *Pe. fluviatilis* (0.014 ± 0.005) values were lower, though insignificant (ANOVA, Tukey HSD post-hoc test, F = 1.9, df = 65.0, *p* > 0.05) (Figure 5b). 

**Figure 6:**
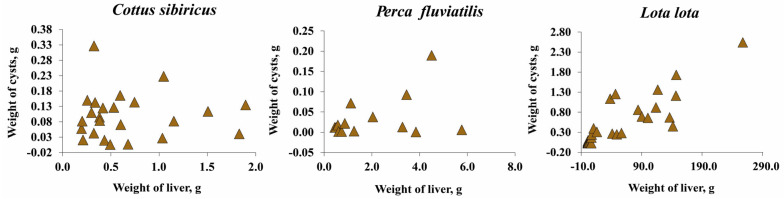
The wet weight of all cysts of *Triaenophorus nodulosus* with weight of liver of different intermediate hosts from Teletskoye Lake.

The total wet weight of *T. nodulosus* plerocercoids did not exceed one percent of the host total body weight and eviscerated body weight of fish (Figure 5c,d; Figure S1). The total wet weight of *T. nodulosus* plerocercoids amounted to 22.8 ± 4.6, 1.9 ± 0.1, and 2.3 ± 0.5 % of the liver weight of *Cot. sibiricus*, *Pe. fluviatilis*, and *L. lota*, respectively (Figure 5c). The weight of individual specimens of *T. nodulosus* plerocercoids amounted to 5.6 ± 1.0, 1.0 ± 0.3, and 0.3 ± 0.1% of the liver weight of *Cot. sibiricus*, *Pe. fluviatilis*, and *L. lota*, respectively (Figure 5d). The scatter plots of all cysts weight to weight of the liver of host are shown in Figure 6.

A positive significant correlation was found between the number of *T. nodulosus* cysts and their total weight as well as to relation of the average individual weight of cysts and number of cysts with the exception of *Cot. sibiricus* (Table 3). 

#### 3.2.3. Definitive Host

The total and individual weight of adult specimens of *T. nodulosus* (0.86 ± 0.23 and 0.14 ± 0.04 g, correspondingly) was significantly higher than *T. crassus* (0.06 ± 0.03 and 0.006 ± 0.004 g, correspondingly) parasitizing the same intestine of *E. lucius* (Figure 5a,b, total weight, ANOVA: F = 12.4, df = 1.0, *p* = 0.008; individual weight, ANOVA: F = 11.0, df = 1.0, *p* = 0.011). The total weight of adult *T. nodulosus* and *T. crassus* amounted to 9.1 ± 0.5 and 2.7 ± 0.2% of the weight of *E. lucius* intestine, respectively. Whereas the weight of individual specimens of adult *T. nodulosus* and *T. crassus* amounted to 1.7 ± 0.05 and 0.8 ± 0.03% % of the weight of *E. lucius* intestine, respectively. 

### 3.3. Clinical Signs of Infestation and Localization of T. nodulosus and T. crassus in Fish

In the case of *T. crassus* plerocercoids, cone-shaped formations under the skin and in the muscles were clearly visible in infected fish. In fish infected with *T. nodulosus* plerocercoids, the liver in several cases was lighter than usual. Sexually mature cestodes caused mechanical damage to the *E. lucius* intestine, accompanied by the growth of connective tissue around the scolexes. In other cases, the fish looked conditionally healthy.

**Figure 7:**
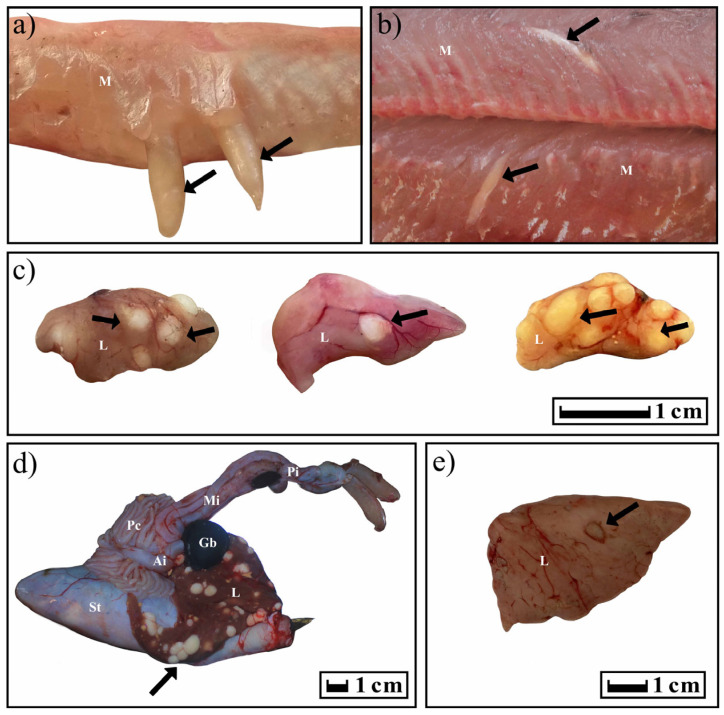
Localization of *T. crassus* and *T. nodulosus* plerocercoids in different intermediate hosts. Black arrows show the localization of cysts. (**a**,**b**) *Cor. l. pidschian*; (**c**) *Cot. sibiricus*; (**d**,**e**) *L. lota*. Abbreviation: St—stomach; L—liver; Ai—anterior intestine; Mi—middle intestine; Pi—posterior intestine; Pc—pyloric caeca; Gb—gallbladder; M—muscles.

The typical distribution of *T. nodulosus* and *T. crassus* plerocercoids in fishes from Teletskoye Lake is shown in Figure 7. For years 2019–2020, atypical localization of *T. nodulosus* plerocercoids was noted on the wall of the intestine (*L. lota*) and body cavity (*Th. arcticus* and *Cot. sibiricus*). In an exceptional case, an immature *T. nodulosus* was observed in the intestine of one single *L. lota*. 

In five cases out of all studied forms/species of *Coregonus lavaretus* whitefishes, an atypical localization of *T. crassus* plerocercoids was observed only for the ‘‘normal” whitefish *Cor. l. pidschian*. Four capsules with plerocercoids were located in the liver, and another one on the surface of the stomach. In the ‘‘normal” whitefish *Cor. l. pidschian* the cysts were more numerous on the right side of the body (*n* = 18) than on the left (*n* = 5), whereas in the “dwarf” whitefish *Cor. l. pravdinellus*, the cysts were more numerous on the left side of the body (*n* = 77), than on the right (*n* = 68). Analysis of symmetry of parasite infestations (binomial exact test) did not reveal any asymmetry (*p* = 0.51) between the number of cysts in the left and right body surfaces of the “dwarf” whitefish *Cor. l. pravdinellus*, whereas in the ‘‘normal” whitefish *Cor. l. pidschian*, an asymmetry of parasite infestations was shown with *p* = 0.011.

On the left and right side of the body of the ‘‘normal” whitefish *Cor. l. pidschian*, the higher number of cysts were located in the hypaxial muscles (*n* = 13) as compared to the epaxial muscles (*n* = 10). In contrast to the ‘‘normal” whitefish *Cor. l. pidschian*, the higher number of cysts on both sides of the body of “dwarf” whitefish *Cor. l. pravdinellus* were located in the epaxial muscles (*n* = 88) in comparison with hypaxial muscles (*n* = 46) and cysts located on the lateral line (*n* = 11) (Table 4).

**Figure 8:**
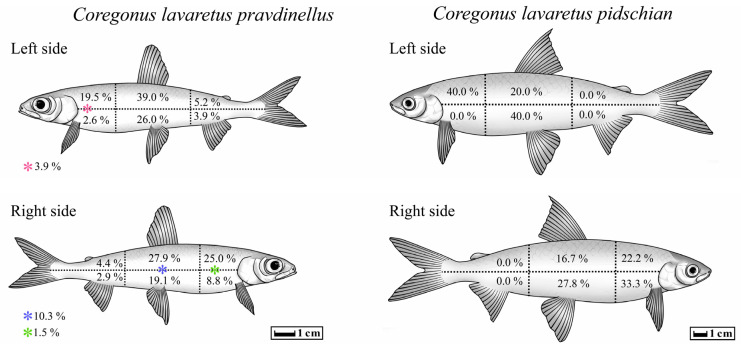
Percentage distribution of *T. crassus* plerocercoids in different parts of the body of different forms/species of whitefishes.

The distribution percentage of the number of cysts in different parts of the body muscles is shown in Figure 8. On the left and right side of the body of both forms/species of whitefishes *Coregonus lavaretus*, the highest percentage values for distribution of the number of cysts were found on the anterior (from head to dorsal fin) and middle (between dorsal fin and adipose fin) part of the fish body. Distribution of cysts in the posterior (tail muscles) of the body were very rare (“dwarf” whitefish *Cor. l. pravdinellus*) or absent (‘‘normal” whitefish *Cor. l. pidschian*). 

## 4. Discussion

The first data associated with diversity of fish parasites in Teletskoye Lake were reported by Titova in 1954 [8]. There were twelve fish hosts for *T. nodulosus* (*Th. arcticus*, *Brachymystax lenok*, *E. lucius*, *L. lota*, *Leuciscus leuciscus*, *Pe. fluviatilis*, *Cot. sibiricus*, *Cot. poecilopus*, *Hucho taimen*, *Cor. lavaretus* sp., *Barbatula toni*, and *Ph. phoxinus*) identified and three host fish for *T. crassus* (*E. lucius*, *L. lota*, and *Cor. lavaretus* sp.) as intermediate and definitive hosts. A relatively high level of prevalence of *T. nodulosus* in the liver was observed for lenok *B. lenok* (85.8%) as the second intermediate host. In the present study, we analyzed the prevalence in liver of the same fish species with the exception of *B. lenok* and taimen *H. taimen* due to these species now being listed in the local Red Book and in need of special conservation attention [16]. In addition, we were not able to analyze the infestation level in *Cot. poecilopus* and *B. toni* due to the absence of these fish species in sites where we set the gill nets. Moreover, according to our results, the common minnow *Ph. phoxinus* was uninfected by *T. nodulosus*, while in the first report [8], a relative high level of prevalence of infestation (up to 66.0%) was found. The contradictions between our results and findings observed early are explained by differences in sites for collection of fish samples. We collected *Ph. phoxinus* from a small river where these fish do not consume zooplankton from Teletskoye Lake, whereas other research investigated *Ph. phoxinus* inhabiting a part of the lake near Yaylyu village (Figure 1b) where planktonic crustaceans were probably much more infescted by *T. nodulosus*. 

**Figure 9:**
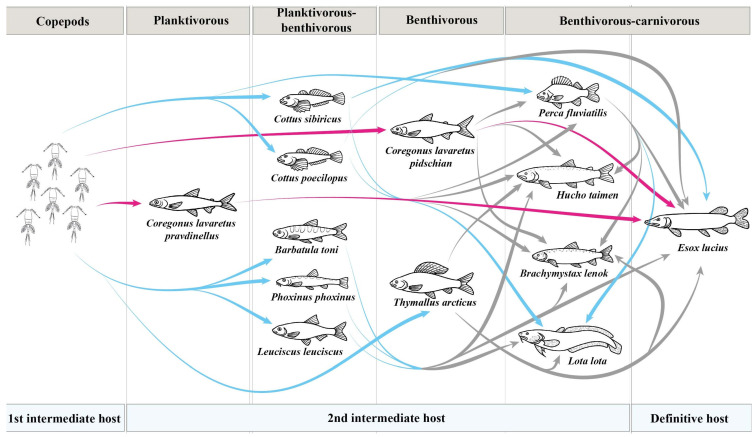
Transmission of *Triaenophorus crassus* and *T. nodulosus* through food webs in Teletskoye Lake. Pink arrow shows the transmission of *T. crassus*, while blue arrow shows the transmission of *T. nodulosus* according to our data and data obtained by Titova, 1954 [8]. Grey arrow indicate a hypothetical rout of infestation.

Based on infestation levels observed, the highest prevalence of *T. nodulosus* infestation in Teletskoye Lake was found for burbot *L. lota* (100%) and is in good agreement with data (93.0%) reported in 1954 [8]. *L. lota* has been reported as a common host for *T. nodulosus* from a wide range of water bodies: Bothnian Bay, Finland (prevalence ranged from 36 to 92%) [17,18]; Baikal Lake, Russia (prevalence 26.7%) [19]; Tsipo-Tsipikan and Kuanda-Chara lakes, Russia (prevalence ranged from 60 to 100%) [20]; etc. We also found *T. nodulosus* in intestines of *L. lota*, but these worms apparently were not able to attach to the intestinal mucosa of *L. lota* and, consequently, were eliminated from the intestine. Other researchers found similar results when immature *T. nodulosus* were observed in *L. lota* intestine [6,17]. Moreover, relatively large *L. lota* do not feed on zooplankton crustaceans, which are the first intermediate hosts for both *Triaenophorus* species, but instead actively feed on strongly planktivorous “dwarf” whitefish–*Cor. l. pravdinellus*. We assume that *L. lota* can be defined as a paratenic host and become infected by *Triaenophorus* larvae when they consume fish whose stomachs are full of infected crustaceous zooplankton (Figure 9). A possible route for the reinvasion of large burbots *L. lota* by *T. nodulosus* plerocercoids is by their penetration from the intestine into the liver with subsequent re-encapsulation, an idea that was also expressed previously [21]. The reinvasion ability of plerocercoids of *Triaenophorus* spp., described as a characteristic feature of this genus, was reported early [22]. From an evolutionary point of view, *T. nodulosus* that infect the liver of *L. lota* never complete their lifecycle due to the adult *L. lota* being one of the largest fish in the lake that cannot be commonly consumed by other carnivorous fish like *E. lucius*, *H. taimen*, and *B. lenok*. Moreover, the illegal fisheries in the lake have eliminated large and valuable individuals of *H. taimen* and *B. lenok*, further reducing pressure on the burbot’s population. At the same time, the young individuals of *L. lota* could be a common prey for other carnivorous fish like *E. lucius* in the lake as was shown for water bodies in northern Finland [17]. 

Among intermediate hosts, the highest prevalence of *T. nodulosus* infestation in Teletskoye Lake was found in the Siberian sculpin *Cot. sibiricus* (prevalence was up to 81.8%). The most common food items of *Cot. sibiricus* in Teletskoye Lake were filamentous algae, larvae of chironomids and Trichoptera, gammarids, molluscs, detritus, and zooplankton. Hence, *Cot. sibiricus* could be directly infected by *T. nodulosus* from the first intermediate host (copepods). At the same time, the ratio of this fish in the *E. lucius* diet was relatively low during the time of sampling (end of August–September, October). Perhaps, *Cot. sibiricus* is an important part of the *E. lucius* diet during other seasons of the year (spring, summer, or winter), but this issue was not the focus of the present study (Figure 9). 

Compared to other second intermediated hosts in Teletskoye Lake, the lower values of prevalence, based on infestation levels of *T. nodulosus*, were shown for perch *Pe. fluviatilis* (24.5%) and arctic grayling *Th. arcticus* (31.9%). The prevalence of *T. nodulosus* in the liver of *Pe. fluviatilis* and *Th. arcticus* were lower almost by half when compared to results of Titova (1954) (65.0% and 76.0%, respectively). Unfortunately, Titova did not describe the size or age groups for *Th. arcticus* and *Pe. fluviatilis* that had been studied, nor the month (it was only noted that it was “summer”) when fish were collected; thus, we cannot explain the differences between hers and our results [8]. *Pe. fluviatilis* is one of the most frequently reported second intermediate hosts for *T. nodulosus* occurring in a wide range of water bodies. Previous studies found that the prevalence level of *T. nodulosus* infestation in *Pe. fluviatilis* from other lakes and rivers was much higher (46.7, 69.9, and 2.9–100%, respectively) than observed in our data [19,23,24]. To a lesser extent, *Th. arcticus* is also mentioned as a second intermediate host. The prevalence of *T. nodulosus* in *Th. arcticus* from Teletskoye Lake was higher (31.9%) than those found in a subspecies of arctic grayling *Th. a. mertensii* (12%) from the Penzhina river (Kamchatsky Krai, Russia) [25]. At the same time, the prevalence level of *T. nodulosus* in *T. a. mertensi* from the Gizhiga River basin (Russia) was 30%, which is in agreement with the level of prevalence found in our study [26]. According to feeding habits, *Pe. fluviatilis* from Teletskoye Lake is a facultative carnivorous fish and generally consumes larvae of chironomids, gammarids, and fish fry, whereas *Th. arcticus* is a benthivorous fish, whose diet includes filamentous algae, larvae of chironomids and Trichoptera, gammarids, mollusks, and stink bugs from the Pentatomidae family. Infestation of these fish occurs as a result of accidental ingestion of copepods in the case of *Th. arcticus*, or as a result of eating fish fry in the case of *Pe. fluviatilis*, in the stomachs of which zooplankton are present in large numbers (Figure 9). 

In Teletskoye Lake, the main second intermediate host for *T. crassus* are sympatric forms/species of whitefishes (“normal” *Cor. l. pidschian* and “dwarf” *Cor. l. pravdinellus*). Unfortunately, Titova [8] did not differentiate these fishes as different forms/species and named all of them as “Teletskoye whitefish”. Titova [8] found that whitefish were more heavily infected by plerocercoids of *T. crassus* at a young age. As shown later, the adult specimens of these whitefishes have many morphological differences [27], but the young ones could be very similar in terms of morphology. Another study demonstrated a significantly higher level of *T. crassus* infestation for “dwarf” *Cor. l. pravdinellus* if compared to “normal” *Cor. l. pidschian* in Teletskoye Lake [28]. Further, Kashinskaya et al. [9] confirmed the findings previously obtained [28]. In the present study, we also found significant differences between infestation levels for forms/species of whitefishes. According to published data [9], the levels of infestation obtained for ‘‘normal” whitefish *Cor. l. pidschian* in the years 2019–2020 were significantly different in comparison with the levels of infestation obtained in 2022 (present study). Thus, the mean intensity and abundance of *T. crassus* infecting the muscles of the ‘‘normal” form/species *Cor. l. pidschian* in 2021–2022 was significantly lower in comparison with previous years [9]. While for the “dwarf” form/species *Cor. l. pravdinellus*, based on collected results over five years (2017; 2019–2022), the infestation levels of *T. crassus* were shown to be relative stable. These differences in levels of infestation between two forms/species of whitefishes were apparently related to feeding habits of these fishes. Indeed, the “dwarf” form/species *Cor. l. pravdinellus* only feeds on zooplankton crustaceans, whereas the diet of the “normal” form/species *Cor. l. pidschian* consists of mollusks, gammarids, detritus, larvae of different aquatic insects, and zooplankton crustaceans that play an insignificant role and perhaps significantly vary from season to season and year to year (Figure 9). 

One of the main aims of the present study was also to estimate the interannual variability of infestation levels of the two species of *Triaenophorus* across the range of common second intermediate and definitive hosts in Teletskoye Lake. Based on results of five years of studies (2017; 2019–2022), we found statistically insignificant interannual variation in the prevalence of *T. nodulosus* in their second intermediate hosts. Our results were also confirmed by a previous study from water bodies in northern Finland, where stability in the infestation level of *T. nodulosus* in different second intermediate hosts was also noted [17]. At the same time, the interannual variation in the prevalence of *T. crassus* in the “normal” form of whitefish *Cor. l. pidschian* was significantly different. For the definitive host (*E. lucius*), the interannual levels of prevalence, intensity, and abundance for *T. crassus* have shown insignificant variations, whereas for *T. nodulosus*, the values were more stable. We assume that the stability in infection levels found in pike of both *Triaenophorus* species is determined by the relative stability of infection levels of their main fish prey (*Cot. sibiricus*, *Pe. fluviatilis*, and whitefishes, as the second intermediate hosts for *T. nodulosus* and *T. crassus*, respectively). 

Moreover, with increasing length and weight of pike, the proportion of *T. crassus* in relation to *T. nodulosus* was also increased and indicated the higher importance of whitefishes in the diet of *E. lucius* with growth. The increased proportion of *T. crassus* in relation to *T. nodulosus* was also reported by other researchers [17,29] from Lesser Slave Lake in Canada and Bothnian Bay in northern Finland. 

In order to study ecological differences among infested fish, we estimated the total, and average weight of the *Triaenophorus* species themselves as well as in relation to the weight of infected organ and body weight of fish. We found that the highest individual weight of cysts and highest ratio of *T. nodulosus* weight to weight of infected organ (liver) was found for *Cot. sibiricus* (0.024 ± 0.003 g and 22.8 ± 4.6%, respectively), whereas the minimum value was for *Pe. fluviatilis* (0.014 ± 0.005 g and 1.9 ± 0.1%, respectively). According to Kuperman [6], the ratio of *T. nodulosus* weight from liver of perch fry ranged from 5.0 to 60.0%. These differences could be related to different immune status of various fish species as well as different levels of infestion. Indeed, the level of *T. nodulosus* intensity in the liver of *Pe. fluviatilis* from Rybinsk Reservoir was much higher (51.8%) when compared to *Pe. fluviatilis* from Teletskoye Lake (26.1% in 2022). 

Moreover, we found a strong positive correlation between the number of worms and their total weight for the majority of studied fish species (*Cot. sibiricus*, *L. lota*, *Pe. fluviatilis*, both forms/species of whitefishes). A positive significant correlation was also found between the average individual weight of cyst (in case of *L. lota*, *Pe. fluviatilis*) and their number. In another study in lakes on Vancouver Island, a positive correlation between the mean size of *Schistocephalus solidus* Muller, 1776 and the parasite’s abundance in threespine stickleback *Gasterosteus aculeatus* Linnaeus, 1758 was also found [30]. It should be considered that the weight of parasites is strongly dependent on their lifespan. Indeed, in cases of *Triaenophorus* species, the cysts in the second intermediate hosts (fishes) could be “young” (from the present year) and “old” (from the previous year), which is reflected in their weight. It can also be related to, for example, different sampling times and characteristics of infestation (how long the fish could be infested by parasites during a year or season) and immune and/or physiological status of the fish. All these parameters are difficult to estimate in nature, hence a similar study but under laboratory conditions is needed where one of the most important parameters—time of infestation—will be controlled.

A typical localization of *T. crassus* plerocercoids is intramuscular, but they may occasionally occur in other organs such as the stomach and pyloric caeca [29]. According to our data, in five cases out of 396 studied whitefishes (2019–2022), the capsules with plerocercoids were located on the liver and the surface of the stomach. In addition, we found that a higher number of cysts in muscles of the ‘‘normal” whitefish *Cor. l. pidschian* was found on the right side of the body when compared to the left one. Similar results were shown for ciscoes *Coregonus artedi* Lesueur, 1818, *Cor. zenithicus* Jordan and Evermann, 1909, *Cor. nipigon* Koelz, 1925, *Cor. nigripinnis* Miller, 1874 from Lesser Slave Lake and Square Lake, Alberta [31]. The author suggested that the greater number of cysts on the right side of the body of coregonids might be explained by the more right-handed location of the stomach and intestine. In a study of asymmetry in the occurrence of eye flukes in *Pe. fluviatilis* Linnaeus, 1758 and *Rutilus rutilus* Linnaeus, 1758 the asymmetrical locations were also seasonal and possibly influenced by the amount of rainfall (likely influencing prey abundance/availability) [32]. The authors attributed the altered left–right distribution to variations in the vasculature of the eye, which is not in disagreement with the idea posited herein regarding the positioning of the stomach and the asymmetry of the helminth infections. Further, Newton [31] studied the distribution of *T. crassus* plerocercoids in whitefish from the same lakes. He found that 80 percent of the cysts were in the epaxial muscles between the head and the dorsal fin. Kuperman [6] also confirmed these data and showed that 82.6% of *T. crassus* plerocercoids infecting *C. albula* Linnaeus, 1758 from Lake Ladoga and Rybinsk Reservoir were in the dorsal muscles, 4.4% on the abdominal wall muscles and 13.0% in the area of anal fin. Similar to Newton [31], we also noted that *T. crassus* plerocercoids in the tail musculature were rare or absent in the case of “dwarf” and ‘‘normal” forms, respectively. This effect can potentially be explained by the fact that penetration of the parasite to tail musculature is restricted by the length of the fish intestine and the parasites’ movement. Indeed, Rosen and Dick [33] also showed that the penetration of parasites more regularly occur through the stomach, pyloric caeca, or anterior intestine. 

## 5. Conclusions

Our study revealed the range of the hosts and levels of infestation of two different *Triaenophorus* species (*T. nodulosus* and *T. crassus*) from a deep, oligotrophic mountain lake in Siberia. Due to the relatively wide range of hosts, we conclude that both *Triaenophorus* species are well integrated into the aquatic system of the lake. Based on the infestation level, the most important hosts in the lifecycle of *T. crassus* and *T. nodulosus* in Teletskoye Lake are *Cor. l. pravdinellus* and *Cot. sibiricus*, respectively. For these fishes, the direct route of parasite transmission to the definitive host was shown. Despite the high level of infestation, *L. lota* in Teletskoye Lake is an evolutionary dead-end host in the lifecycle of *T. nodulosus* due to it occupying the niche of the largest carnivorous fish. An indirect route of *Triaenophorus* spp. transmission in Teletskoye Lake has been shown for the “normal” *Cor. l. pidschian*, *Th. arcticus*, and facultative carnivorous *Pe. fluviatilis*. Infestation of these fishes may occur as a result of accidental ingestion of copepods or reinvasion by *Triaenophorus* spp. This assumption confirms our first hypothesis. Based on results from five years of sampling, we found stability of *T. nodulosus* in their second intermediate hosts, whereas for *T. crassus* infestations, they show significant interannual variation. At the same time, for the definitive host (*E. lucius*), the interannual levels of prevalence, intensity, and abundance for *T. nodulosus* were more stable, whereas for *T. crassus* there were only insignificant variations to confirm our second hypothesis. Differences in feeding habits and physiology of various fishes probably may affect on the mass of parasites through their abundance, supporting our third hypothesis but needs more extensive studies in the future. At the same time, the high rates of invasion by *Triaenophorus* plerocercoids can potentially affect the locomotor activity of fish. The lower activity of the host under parasitic invasion makes the fish more accessible to the definitive host, which, as a result, increases the success of implementation of the parasite lifecycle.

## Figures and Tables

**Table 1:** Summary data of total body weight (BW), eviscerated body weight (EBW), total length (TL), sex ratio, and fish diet of seven fish species examined for presence of *Triaenophorus crassus* and *T. nodulosus* from Teletskoye Lake through different years. * F—female, M—male.

Fish Species	Fish Diet in Teletskoye Lake	Stage in Fish	Localization	Parasite Species	Year	Number Fish Examined	BW, g (Mean ± SE)	EBW, g (Mean ± SE)	TL, mm (Mean ± SE)	Sex Ratio (F:M) *
*Coregonus lavaretus pidschian* ^1^	Chironomids (larvae), gammarids, mollusks (gastropoda)	Larvae	Muscles	*T. crassus*	2021	41	176.3 ± 17.0	-	266.6 ± 7.6	1.7:1
					2022	99	160.6 ± 10.6	140.3 ± 9.4	247.9 ± 5.0	1:2.3
*Coregonus lavaretus pravdinellus* ^2^	Zooplankton	Larvae	Muscles	*T. crassus*	2021	21	31.0 ± 1.3	25.5 ± 1.5	167.5 ± 1.7	1:1.3
					2022	68	33.8 ± 0.8	27.5 ± 0.8	172.5 ± 2.2	1:4.7
*Cottus sibiricus* ^3^	Filamentous algae, zooplankton, chironomids (larvae), gammarids, mollusks (gastropoda), Trichoptera (larvae), detritus	Larvae	Liver	*T. nodulosus*	2019	4	-	-	-	-
					2020	12	12.0 ± 1.1	11.9 ± 1.2	92.8 ± 3.1	1:2
					2021	11	20.4 ± 3.1	-	117.8 ± 4.2	0:1
					2022	28	14.4 ± 1.2	11.9 ± 0.9	108.0 ± 2.6	1:1.2
*Perca fluviatilis* ^4^	Chironomids (larvae), gammarids, fish fry	Larvae	Liver	*T. nodulosus*	2019	20	-	-	-	-
					2020	22	138.4 ± 15.0	119.2 ± 12.9	216.7 ± 7.7	1:4.3
					2021	14	257.4 ± 13.7	222.8 ± 9.9	264.5 ± 3.4	12:1
					2022	46	117.9 ± 16.1	100.6 ± 12.2	195.4 ± 8.3	1.2:1
*Thymallus arcticus* ^5^	Filamentous algae, chironomids (larvae), gammarids, mollusks (gastropoda), Trichoptera (larvae)	Larvae	Liver	*T. nodulosus*	2021	24	328.7 ± 40.8	312.3 ± 40.8	306.9 ± 11.1	1.4:1
					2022	23	160.0 ± 15.7	143.0 ± 13.9	250.7 ± 7.4	1.1:1
*Lota lota* ^6^	Filamentous algae, chironomids (larvae), gammarids, mollusks (gastropoda), fish fry	Larvae	Liver	*T. nodulosus*	2021	5	804.3 ± 356.6	678.7 ± 314.0	487.3 ± 47.2	2:1
					2022	30	728.0 ± 175.1	603.3 ± 143.7	377.9 ± 36.0	1.8:1
*Phoxinus phoxinus* ^7^	Filamentous algae, mollusks (gastropoda), zooplankton, larvae of insects	Larvae	Liver	*T. nodulosus*	2022	8	1.39 ± 0.07	-	57.88 ± 0.81	-
*Esox lucius* ^8^	Fish	Adult	Intestine	*T. crassus*, *T. nodulosus*	2021	17	467.3 ± 76.1	410.0 ± 64.3	405.7 ± 23.7	1:2.5
					2022	14	519.7 ± 141.8	474.4 ± 124.3	418.0 ± 29.2	

Abbreviation names: 1—*Cor. l. pidschian*; 2—*Cor. l. pravdinellus*; 3—*Cot. sibiricus*; 4—*Pe. fluviatilis*, 5—*Th. arcticus*; 6—*L. lota*; 7—*Ph. phoxinus*; 8—*E. lucius*.

**Table 2:** Infestation indices of *T. crassus* and *T. nodulosus* in different intermediate and definitive hosts from Teletskoye Lake.

Fish Species	Parasite	Year	Prevalence, %	Intensity (Mean ± SE)	Abundance (Mean ± SE)	Total Number of Fish	Number of Healthy Fish	Number of Fish Infected	Number of Parasites
*Cor. l. pidschian*	*T. crassus*	2021	41.5	1.8 ± 0.4	0.8 ± 0.2	41	24	17	31
		2022	27.3	1.8 ± 0.5	0.5 ± 0.1	99	72	27	50
		2021–2022	31.4	1.8 ± 0.2	0.6 ± 0.1	140	96	44	81
*Cor. l. pravdinellus*		2021	100.0	5.0 ± 0.8	5.0 ± 0.8	21	0	21	105
		2022	89.7	4.5 ± 0.5	4.0 ± 0.5	68	7	61	274
		2021–2022	92.1	4.6 ± 0.4	4.3 ± 0.4	89	7	82	379
*E. lucius*		2021	64.7	23.6 ± 6.3	15.3 ± 8.7	17	6	11	260
		2022	78.6	12.3 ± 5.6	9.6 ± 3.8	18	7	11	135
		2021–2022	71.0	17.9 ± 5.7	12.7 ± 5.0	35	13	22	395
*Cot. sibiricus*	*T. nodulosus*	2019	100.0	5.0 ± 1.5	5.0 ± 1.5	4	0	4	20
		2020	66.7	3.6 ± 0.8	2.4 ± 0.7	12	4	8	29
		2021	100.0	8.0 ± 2.3	8.0 ± 2.3	11	0	11	88
		2022	78.6	4.2 ± 0.6	3.3 ± 0.6	28	6	22	93
		2019–2022	81.8	5.1 ± 0.7	4.2 ± 0.6	55	10	45	230
*Pe. fluviatilis*		2019	20.0	1.2 ± 0.2	0.2 ± 0.1	20	16	4	5
		2020	27.3	1.3 ± 0.2	0.4 ± 0.1	22	16	6	8
		2021	21.4	1.0 ± 0.0	0.2 ± 0.1	14	11	3	3
		2022	26.1	1.7 ± 0.2	0.5 ± 0.1	46	34	12	21
		2019–2022	24.5	1.5 ± 0.1	0.4 ± 0.1	102	77	25	37
*Th. arcticus*		2021	33.3	4.1 ± 1.3	1.4 ± 0.6	24	16	8	33
		2022	30.4	2.6 ± 0.3	0.8 ± 0.1	23	16	7	18
		2021–2022	31.9	3.4 ± 1.1	1.1 ± 0.3	47	32	15	51
*L. lota*		2021	100.0	7.4 ± 2.2	7.4 ± 2.2	5	0	5	37
		2022	100.0	23.5 ± 3.7	23.5 ± 3.7	30	0	30	706
		2021–2022	100.0	21.2 ± 3.3	21.2 ± 3.3	35	0	35	743
*E. lucius*		2021	76.5	18.8 ± 4.8	14.3 ± 4.1	17	4	13	244
		2022	78.6	19.7 ± 6.7	15.5 ± 6.3	18	7	11	217
		2021–2022	77.4	19.2 ± 3.7	14.9 ± 3.6	35	11	24	461

**Table 3:** Relationship between the wet weight of *T. crassus* and *T nodulosus* and number of cysts in different intermediate hosts from Teletskoye Lake.

Species	Parasite	Mean Weight of all Cysts and Number of Cysts		Average Individual Weight of Cyst and Number of Cyst	
		Spearman’s r	*p*-Value	Spearman’s r	*p*-Value
*Cor. l. pravdinellus*	*T. crassus*	0.70	<0.001	−0.10	0.446
*Cor. l. pidschian*		0.70	<0.001	0.19	0.261
*Cot. sibiricus*	*T nodulosus*	0.78	<0.001	0.01	0.972
*Pe. fluviatilis*		0.87	<0.001	0.67	0.009
*L. lota*		0.90	<0.001	0.36	0.054

**Table 4:** Localization and percent distribution of *T. crassus* plerocercoids in muscles of forms/species of whitefishes *Coregonus lavaretus*.

Forms/Species	Distribution	In the Middle of Epaxial and Hypaxial Muscles	Epaxial Muscles	Hypaxial Muscles	Epaxial Muscles	Hypaxial Muscles	Epaxial Muscles	Hypaxial Muscles	Total
			From Head to Dorsal Fin (I)		Between Dorsal Fin and Adipose Fin (II)		Tail Muscles (III)		
“Dwarf” *Cor. l. pravdinellus*	*Left side of body*								
	Number of cysts	3	15	2	30	20	4	3	77
	%	3.90	19.48	2.60	38.96	25.97	5.19	3.90	100.00
	*Right side of body*								
	Number of cysts	8	17	6	19	13	3	2	68
	%	11.76	25.00	8.82	27.94	19.12	4.41	2.94	100.00
	*Both sides of body*								
	Number of cysts	11	32	8	49	33	7	5	145
	%	7.60	22.07	5.52	33.79	22.76	4.83	3.45	100.00
“Normal” *Cor. l. pidschian*	*Left side of body*								
	Number of cysts	-	2	0	1	2	0	0	5
	%	-	40.00	0.00	20.00	40.00	0.00	0.00	100.00
	*Right side of body*								
	Number of cysts	-	4	6	3	5	0	0	18
	%	-	22.22	33.33	16.67	27.8	0.00	0.00	100.00
	*Both sides of body*								
	Number of cysts	-	6	6	4	7	0	0	23
	%	-	26.09	26.09	17.39	30.43	0.00	0.00	100.00

## Data Availability

All data generated or analyzed during this study are included in this published article.

## Supplementary Materials

The following supporting information can be downloaded at: https://www.mdpi.com/article/10.3390/ani13193122/s1, Figure S1: Size of cestodes (white bar) and their relationship with eviscerated body weight of fish (blue bar).
