# Supplementary figures and images for: Metapopulation Structure of Two Species of Pikeworm (Triaenophorus, Cestoda) Parasitizing the Postglacial Fish Community in an Oligotrophic Lake
Source: Animals (Basel). 2023 Oct 6;13(19):3122. doi: 10.3390/ani13193122 (PMC10571662; doi:10.3390/ani13193122)

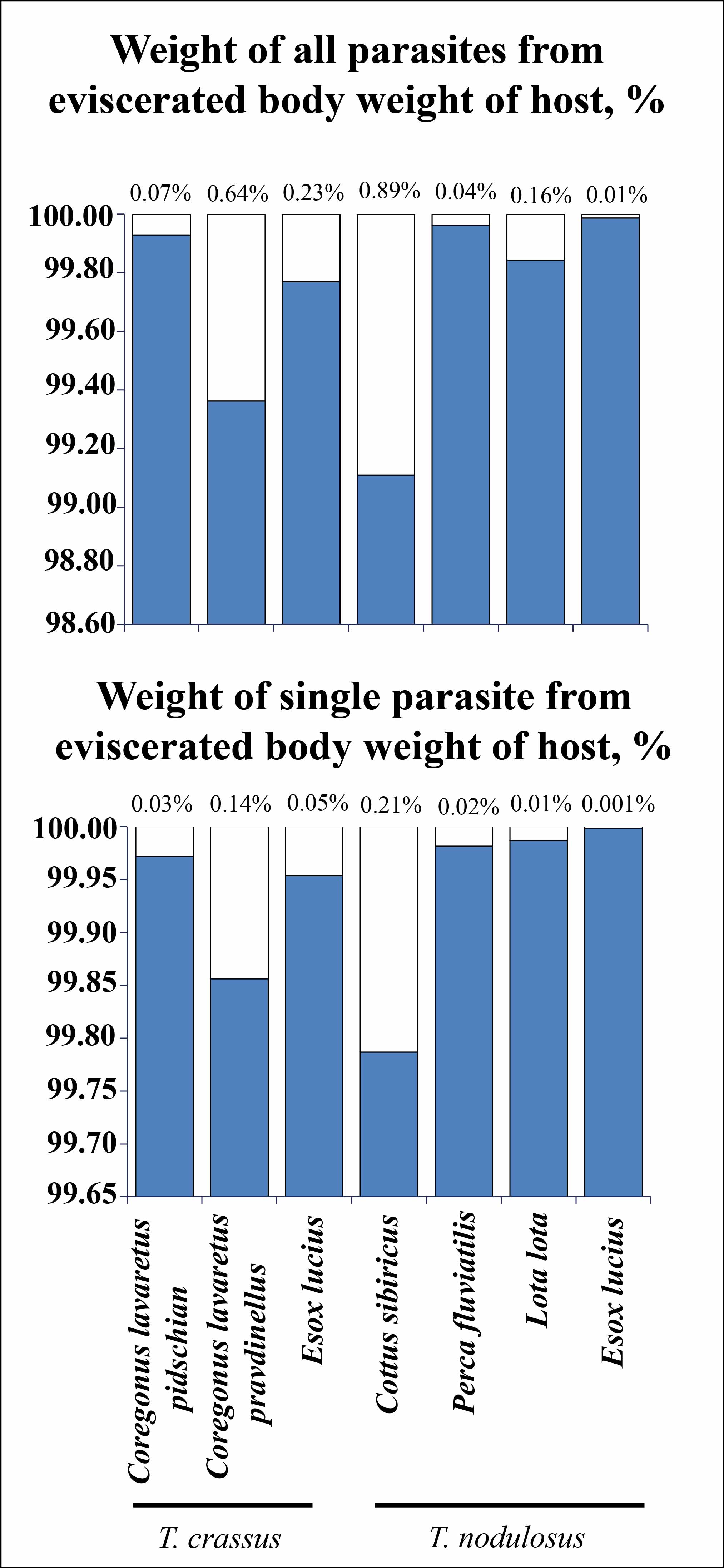

Supplement: Supplementary file 1 [file animals-13-03122-s001.zip › animals-2502387-supplementary.jpg]
